# A New Approach for Orbital Wall Reconstruction in a Rabbit Animal Model Using a Hybrid Hydroxyapatite–Collagen-Based Implant

**DOI:** 10.3390/ijms252312712

**Published:** 2024-11-26

**Authors:** Victor A. Vasile, Sinziana Istrate, Laura-Madalina Cursaru, Roxana M. Piticescu, Aurelian M. Ghita, Diana M. Popescu, Gerhard Garhöfer, Ana M. Catrina, Sonia Spandole-Dinu, Cerasela Haidoiu, Vladimir Suhaianu, Oana C. Voinea, Dumitru Valentin Dragut, Alina Popa-Cherecheanu

**Affiliations:** 1 Department of Ophthalmology, Faculty of Medicine, Carol Davila University of Medicine and Pharmacy, 020021 Bucharest, Romania; 2 Department of Ophthalmology, Ophtalmopôle, Hôpital Cochin, AP-HP, 75014 Paris, France; 3 BINE Ophthalmology Clinic, 020483 Bucharest, Romania; 4 Nanostructured Materials Laboratory, National R&D Institute for Nonferrous and Rare Metals, 077145 Pantelimon, Romania; 5 Department of Ophthalmology, Emergency University Hospital, 050098 Bucharest, Romania; 6 Cantacuzino National Military Medical Institute for Research and Development, 050096 Bucharest, Romania; 7 Department of Clinical Pharmacology, Medical University of Vienna, 1090 Vienna, Austria; 8 Department of Pathology, Faculty of Medicine, Carol Davila University of Medicine and Pharmacy, 020021 Bucharest, Romania

**Keywords:** orbital reconstruction, orbital implants, hydroxyapatite, 3D-printed structures, hybrid coatings

## Abstract

Reconstructing the orbit following complex craniofacial fractures presents significant challenges. Throughout the years, several materials have been used for orbital reconstruction, taking into account factors such as their durability, compatibility with living tissue, cost efficiency, safety, and capacity to be adjusted during surgery. Nevertheless, a consensus has not yet been reached on the optimal material for orbital restoration. This study investigates the potential of a hybrid hydroxyapatite–collagen (HAp-COL) material 3D-printed on Ti mesh to be used as an implant for orbital wall reconstruction. HAp-COL powder was synthesized using a high-pressure hydrothermal technique. The powder was further used to 3D-print HAp-COL structures on titanium mesh, with the latter having potential uses in orbital wall reconstruction. Biocompatibility was assessed by evaluating the effects of the HAp-COL material on the adhesion and proliferation of fibroblasts (3T3) and mesenchymal stem cells (MSCs) in culture. In vitro and in vivo results showed that HAp-COL is highly biocompatible and has a good integration of the implant in the bone. The findings reported in this study offer convincing evidence to support the use of our designed HAp-COL for the restoration of orbital wall fractures, with a high level of safety.

## 1. Introduction

Orbital fractures are frequently encountered in cases of midface trauma [1]. The orbits, characterized by their conical shape and irregular walls, protect the eyeballs and delineate the boundary between the upper and middle facial skeleton. In adults, these fractures are primarily caused by domestic violence and motorcycle accidents, while in children, sports injuries and falls are the most common causes [2]. Various factors such as social characteristics, demographics, study period, and cultural context related to facial trauma shape the epidemiological profile of patients with facial fractures [3,4]. A Swedish study conducted from 1986 to 1996 identified road traffic accidents as the leading cause of this injury [5], while in a study involving soldiers in the US Army, assault was identified as the most common cause of this injury [6].

The craniofacial region is anatomically complex due to its numerous interconnected elements, each with varying degrees of resistance or vulnerability to impacts based on their unique structural and biomechanical properties. Consequently, treating injuries in this area requires a tailored approach that focuses on restoring its morphology while maintaining the functions and connections with the skull vault and base above and behind, as well as the mandible below, to achieve a cohesive cranial structure [7,8]. While the fundamental concepts of managing these injuries have remained largely unchanged over the years, significant advancements have been made. These include improvements in maxillofacial and orbital imaging, the introduction of intraoperative navigation systems, more robust evidence-based surgical indications and timing, and enhanced implant designs. As a result, traditional techniques and guidelines have been re-evaluated and refined [9]. A variety of implants are available for orbital reconstruction, each offering unique characteristics that are selected based on the surgeon’s assessment of factors such as the specifics of the fracture, the patient’s age, and the affected area. Traditionally, autografts were the preferred option for this procedure. However, advancements in material science and biocompatibility have led to the predominance of alloplastic implants in contemporary orbital reconstruction [10]. Over the past decade, various individual implants such as high-density polyethylene (HDPE), titanium, hydroxyapatite (HAp), polydioxanone, and polylactic acid/polyglycolic acid have been utilized for orbital fracture reconstruction [11]. These therapeutic approaches show great promise due to their potential to yield excellent ophthalmologic outcomes. However, no implant has yet been developed that fully replicates the functional properties of natural bone while supporting cell proliferation and anchorage [12]. Meeting these criteria is essential for achieving osseointegration and ensuring the optimal biomechanical performance of the bone–implant interface [13]. The materials used in bone implantation must promote a specific interaction with the internal environment of the human body and must exhibit certain mechanical, physicochemical, and biological qualities. The most essential characteristic among these is biocompatibility [14].

Hydroxyapatite is recognized for its non-toxic, non-inflammatory, and non-immunogenic characteristics, meaning that it is often integrated into polymeric composites for bone tissue engineering purposes [15]. The use of bioactive materials, such as hydroxyapatite coatings on metallic implants, has garnered significant attention due to their chemical composition and crystallographic structure, which closely mimic the minerals found in human bones and teeth. The strong chemical bond formed between HAp coatings and implant surfaces enhances the growth of new bone tissue. Numerous topographical and physicochemical surface modifications have been established, encompassing additive techniques like titanium plasma spray (TPS) and hydroxyapatite (HA) coatings, as well as subtractive techniques including electropolishing, mechanical polishing, acid treatments, sandblasting, oxidation, and laser irradiation. These microtopographic alterations have repeatedly shown favorable outcomes in augmenting osseointegration metrics, including elevated removal torque values and enhanced bone quantity and quality [16]. As a result, HAp-coated alloys are expected to overcome the limitations of biocompatible metals while addressing the brittleness and inferior mechanical properties often associated with HAp coatings [17]. Numerous studies on titanium implants coated with HAp have highlighted several biological advantages. These benefits include enhanced bone formation on the surface of the thin hydroxyapatite layer, robust attachment at the interface between the HAp coating and bone, improved bone ingrowth into the pores of HAp-coated metallic implants, and protection of the surrounding bone from metal ion release from the implant substrate. Compared to uncoated implants, HAp coatings on metal implants demonstrate a superior ability to bind with bone and exhibit strong osteoconductive properties [18].

In 1985, Brånemark introduced the term “osseointegration” to define the direct, microscopic-level connection between living bone and a load-bearing endosseous implant [19]. One strategy to enhance osseointegration involves precoating implants with extracellular matrix proteins to facilitate targeted interactions between cells and the matrix. Additionally, applying growth factors to the implant surface promotes the differentiation of osteoblasts. Proteins such as collagen types I and IV, solubilized elastin, vitronectin, and fibronectin have proven effective in enhancing cellular adhesion and spreading. These proteins contain specific sequences, known as RGD (arginine–glycine–aspartic acid), which are recognized by integrins, thereby promoting cell attachment [20]. Type I collagen has been selected as a coating for implant surfaces for several reasons. Firstly, it offers high dimensional stability, which is crucial for developing the extracellular matrix (ECM). Additionally, type I collagen is a key component of both hard and soft tissues, making it an ideal choice. As the most abundant protein in the human body, collagen is vital for tissue repair and regeneration. Specifically, type I collagen, mainly produced by osteoblasts, is plentiful in bone tissue and serves as a scaffold for new bone formation. It primarily binds to integrins alpha1beta1 and alpha2beta1, influencing the adhesion and differentiation of osteoblastic cells [21].

Human bone is a highly vascularized, dynamic tissue that continues to develop, regenerate, and function throughout an organism’s lifetime. It may react to a wide range of stimuli and is in charge of many functions (e.g., metabolic, physical, and endocrine) [22]. Bone consists of around 70% inorganic material, 20% organic matrix, and 10% water. The primary component of bone mineral is HAp, whereas the major constituent of the organic matrix is type I collagen, accounting for around 90% of its composition. Additionally, non-collagenous proteins (NCPs) such as osteonectin and osteocalcin make up around 10% of the matrix [23,24]. Biomechanically, the inorganic mineral content, primarily hydroxyapatite, provides bone with its rigid structural framework, while collagen imparts its elastic properties, allowing for flexibility and resilience [24,25]. To replicate the natural composition of bone, scaffolds for bone tissue engineering could be composed of hydroxyapatite–collagen (HAp-COL) hybrid materials.

Numerous literature studies have focused on investigating the biological properties of compounds based on hydroxyapatite and collagen. Thus, J. Kozlowska and collaborators [14] used both in vitro and in vivo methods to investigate the biological properties of porous collagen–hydroxyapatite matrices (80/20), obtained through the freeze-drying method followed by a dehydrothermal treatment (DHT, 110 °C, under vacuum, 24 h) for stabilization. Mouse fibroblast cells were used for in vitro tests, and in vivo studies were performed through subdermal implantation in the peritoneal cavity in rats for 30 days. The structures were well tolerated by the cells, which was confirmed by the results of in vitro cytotoxicity tests. Also, the rate of vascularization and incorporation into the host tissue is substantially increased due to the conditions of the extracellular matrices, material components, and 3D design. The absence of necrosis or abscess formation in the surrounding tissues demonstrates good biocompatibility as well as low local irritability. Preliminary histological studies have demonstrated vascularization of the implant 30 days after implantation [26]. Therefore, the collagen–hydroxyapatite hybrid material can find new applications in the field of tissue engineering.

Knowing that bones are capable of regeneration, it must be taken into account that bone defects must be filled with a porous structure that allows both tissue growth and the deposition of the extracellular matrix.

Although bones have an intrinsic capacity for recovery, repairing their critical defects, caused by tumors, traumas, and infections, is a major challenge [27]. Bone regeneration by applying allografts and autografts is restricted due to several factors, such as limited availability, complications that can occur due to the donor area, as well as the risk of disease transmission [28,29]. Thus, the development of three-dimensional (3D) structures for the purpose of bone regeneration has become a key factor in bone tissue engineering [30].

Three-dimensional (3D) printing techniques, also referred to as additive manufacturing (AM) processes, are considered emerging technologies. Since their invention in 1980, they have seen continuous development, maturation, and use by a large number of researchers and industrial companies worldwide in various applications [31,32].

Among 3D printing methods, robocasting or direct ink writing (DIW) is an additive manufacturing technique defined as the method by which a material, in the form of a paste, is extruded layer by layer through a nozzle until the previously designed structure is obtained in a CAD software (e.g., SolidWorks 2019) [33]. Among the advantages of using this technique are the lower cost of devices and raw materials, the adaptability and variability of the materials that can be printed (compared to the requirements of other AM technologies, such as narrow particle size distribution, excellent fluidity, specific light absorption, etc. [34]). Moreover, unlike other AM methods, robocasting can obtain 3D-printed structures at room temperature without the need for lasers or ultraviolet light, and the quality of the deposition paste depends only on its rheological properties that can be controlled during its preparation [35].

Thus, the growing trend of adopting the additive manufacturing technique to the detriment of traditional methods is based on several important advantages such as obtaining complex geometries with high precision, material economy, flexibility, and customized design. However, the limited number of materials available is a great disadvantage of the method, which is why, in recent years, the attention of researchers has turned to the development of new materials suitable for additive manufacturing processes [35].

In recent years, there has been growing interest in the use of 3D modeling in the medical field, due to the provision of efficient solutions for several applications [36,37].

Initially, these techniques were considered rapid prototyping techniques. At present, due to the increasing demand for both complexity and multifunctionality, these techniques are revolutionizing manufacturing by exploiting many smart materials, biomaterials, nanomaterials, composite materials, hybrid materials, etc. [32,38].

All AM processes work on the principle of successive addition of layers, for the construction of porous, biocompatible structures of predefined shapes, with excellent mechanical and osteoconductive properties [31,36].

Thus, traditional implant manufacturing processes have become outdated, with the survival rate of implants produced using AM being improved by two times. Three-dimensional printing techniques offer the possibility of printing bone substitute materials with a complex shape, chemistry, porosity, and well-controlled interior/exterior architecture with interconnectivity, which allow vessels and nerves to develop easily, enhancing the growth of bone cells and tissues [37,39,40].

In this context, one potential application of 3D-printed HAp-COL hybrid material could be the reconstruction of orbital wall fractures. We present the synthesis and processing through extrusion-based 3D printing (robocasting) of a hydroxyapatite–collagen hybrid material for the reconstruction of orbital wall fractures. As a novelty, this paper presents a new approach for HAp-COL coatings with potential use as orbital implants: 3D structures based on HAp-COL hybrid powders (prepared using a hydrothermal method) are deposited layer by layer using a robocasting technique—an emergent 3D printing technique—directly on Ti mesh. To our knowledge, this is the first study to explore the use of a 3D-printed structure based on HAp-COL hybrid material, coated on a titanium mesh, specifically for this application. The originality of the solution proposed in this study consists of combining the properties of 3D-printed porous structures with those of Ti mesh, due to the versatility of the robocasting technique, which enables the personalized 3D printing of HAp-COL strictly on Ti surfaces of the mesh. The objective of this paper is to characterize engineered hydroxyapatite–collagen hybrid materials in terms of their physicochemical properties, in vitro behavior, and interactions with living systems for the purpose of reconstructing orbital wall fractures.

## 2. Results and Discussion

### 2.1. Physicochemical, Structural, and Morphological Characterization of the Hybrid Materials

#### 2.1.1. Quantitative Chemical Analysis of the Hybrid Powder

The Ca and P content of the investigated hybrid powder is shown in Table 1.

The molar ratio of Ca:P content obtained from the chemical analysis of hydroxyapatite–collagen hybrid powder is ~1.68, while the theoretical ratio is Ca:P = 1.67, which confirms the formation of hydroxyapatite.

#### 2.1.2. FT-IR Spectroscopy Analysis of the Hybrid Powder

**Figure 1:**
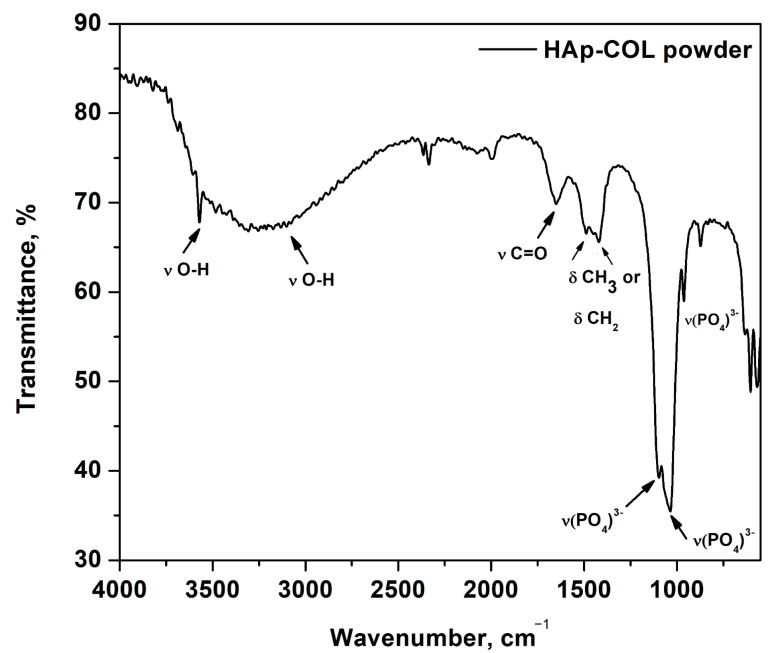
FT-IR spectrum of HAp-COL sample.

The characterization of the HAp-COL sample through Fourier transform infrared spectrometry (FT-IR) revealed the presence of the following vibration bands, which are characteristic for hydroxyapatite: (i) the stretch vibration of the OH group (sharp band) from 3550 cm^−1^; (ii) the stretch vibration of the water molecule (3034 cm^−1^), large band; (iii) the stretch vibrations of the (PO_4_)^3−^ group from 1095, 1036, and 962 cm^−1^. The existence of collagen was evidenced by the presence of vibration bands of the CH_2_ and CH_3_ groups from 1487 and 1420 cm^−1^ (deformation vibrations) and the amide I band (νC = O) from 1649 cm^−1^ (Figure 1) [41,42,43].

#### 2.1.3. X-Ray Diffraction Analysis of the Hybrid Powder

**Figure 2:**
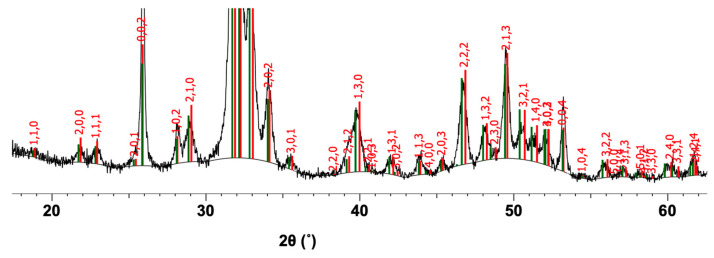
Graphical presentation of qualitative phase analysis using XRD for hybrid powder.

The main crystalline phases identified in the hybrid powder obtained through hydrothermal synthesis, as well as the crystallization systems of these crystalline phases, are described in Table 2. The diffraction spectrum is shown in Figure 2.

From Figure 2, it can be seen that the main crystalline phase is hydroxyapatite 2, with the chemical formula Ca_5_(PO_4_)_3_(OH), PDF 00-071-5048, with a hexagonal crystallization system.

##### Rietveld Semi-Quantitative Analysis

In order to perform the Rietveld analysis, the two identified structures were used to perform a better fit of the collected experimental profile. The analysis was carried out using the Bruker Suite Topas v.5 software.

The Rietveld semi-quantitative analysis (S-Q, %), used to determine the phase percentages present in the HAp-COL sample, is based on the assumption that all phases present are crystalline and are included in the analysis. The method is based on the calculation of the weight ratio defined according to the following equation:

$$W_{\alpha }=\frac{S_{\alpha }{\left(ZMV\right)}_{\alpha }}{\sum_{j=1}^{n}S_{j}{\left(ZMV\right)}_{j}}$$

where S_α_ is the scaling factor of the α phase fraction; ZM represents the mass of the contents of the elementary cell; V is the volume of the elementary cell; and n is the number of phases present in the analysis [44]. The calculation of the crystallite sizes was performed using the equation proposed by Scherrer, with a modification made by Laue, by which the integral width of the diffraction maxima is taken into account. By using the integral width in this calculation, an independent evaluation of the distribution of sizes and shapes is obtained [44]:

$$D=\frac{\lambda }{\beta_{IB}cos\theta }$$

Thus, for the two identified structures, the values of the crystallite sizes are as follows:

Hexagonal structure hydroxylapatite 1: ~6.37 μm.

Hexagonal structure hydroxylapatite 2: ~17 nm.

A difference in the average crystallite size between the two crystalline phases is observed. Thus, the majority phase (hydroxyapatite 2) has a crystallite size in the nanometer range, while the second phase (hydroxyapatite 1) has crystallite sizes in the micron range.

#### 2.1.4. Electron Microscopy (SEM) Analysis and EDS Semi-Quantitative Analysis of the Hybrid Powder

**Figure 3:**
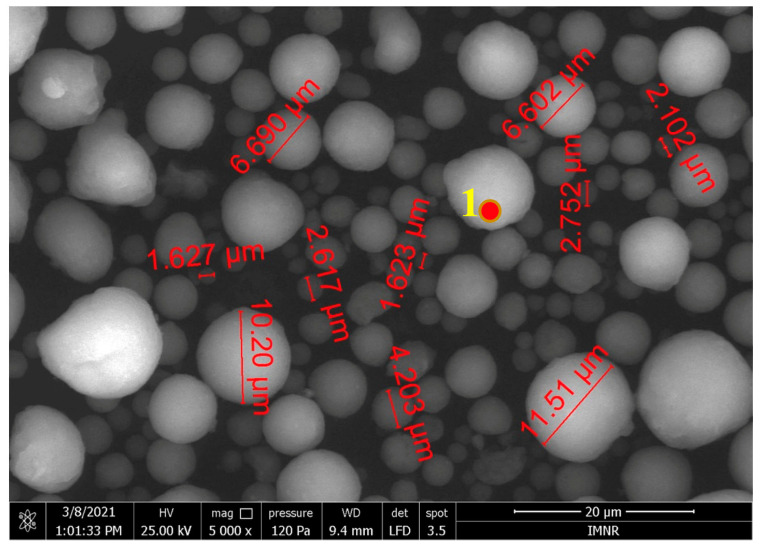
SEM micrograph of the hybrid powder, obtained with the large-field secondary-electron detector.

Electron microscopy has highlighted the spheroidal character of the powder, observing dimensions ranging up to tens of microns (Figure 3). The spheroidal shape of the particles is due to spray-drying and favors the formation of homogeneous pastes (based on HAp-COL hybrid powder and commercial dispersants) with good flow properties for the manufacture of three-dimensional structures [45].

**Figure 4:**
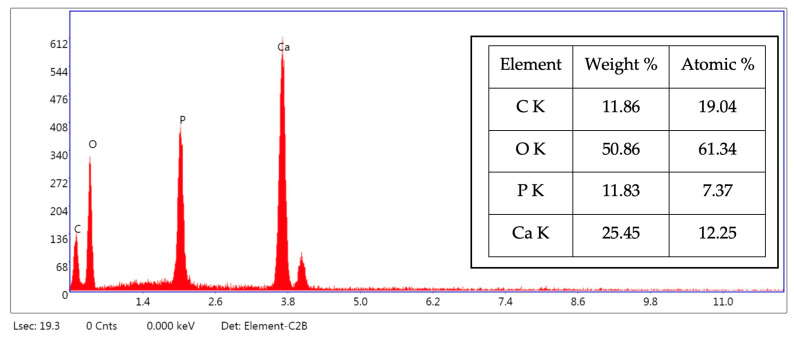
EDS spectrum of HAp-COL hybrid powder in point 1 from Figure 3.

Additionally, EDS characterization (Figure 4) shows the presence of Ca and P in the hybrid powder with a ratio of 1.67, which confirms the results of the chemical analysis (Table 1).

#### 2.1.5. Electron Microscopy (SEM) Analysis and EDX Semi-Quantitative Analysis of the Hybrid 3D-Printed Structures

**Figure 5:**
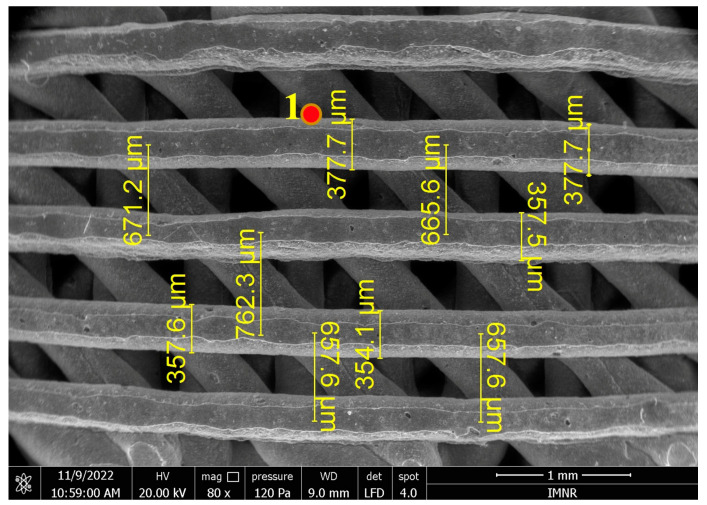
EDS-SEM micrograph of a stand-alone hybrid 3D-printed structure, obtained using the large-field secondary-electron detector.

Three-dimensional structures obtained through an additive manufacturing technique, as described in Section 2.1, have been characterized using the SEM-EDS method to determine the strand thickness as well as the distance between strands, as observed in Figure 5.

**Figure 6:**
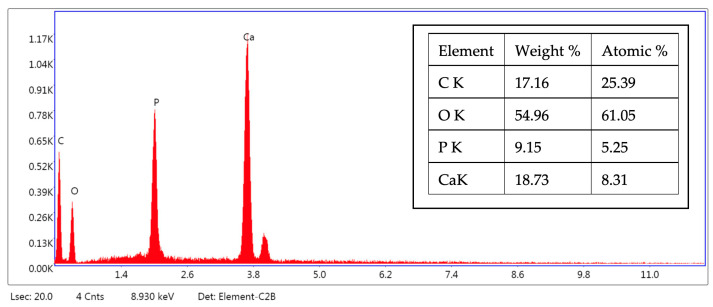
EDS spectrum of a stand-alone hybrid 3D-printed structure in point 1 from Figure 5.

From Figure 5, it can be seen that both the strand thickness obtained after 3D printing (354–377 μm) and the distance between strands (657–762 μm) are in accordance with the values set in the printer software (thickness = 400 μm and distance between strands = 800 μm), with a slight contraction that may be due to the drying process. The semi-quantitative point chemical analysis EDS highlighted the presence of the following elements: O, C, Ca, and P. The atomic and mass percentages were related to the analyzed point presented in the table in Figure 6. The Ca:P molar ratio is about 1.59, due to the presence of organic additives (dispersants and binders).

### 2.2. Evaluation of Biocompatibility Using In Vitro Cell Culture Studies

Williams described biocompatibility as the ability of a device to perform its intended function within the host, achieving the desired level of integration without causing any adverse local or systemic effects in the host [46]. Biocompatibility is a critical requirement for any biomaterial, indicating that the introduction of the material does not induce cell death or interfere with the normal functioning of cells and tissues. Biocompatible materials, whether inorganic or organic, are capable of being implanted into the human body to replace or repair damaged tissues. These materials, referred to as biomaterials, are specifically designed to interact directly with the body and its various biological systems [47].

Biomaterials must demonstrate both biosafety and biostability, ensuring they are non-toxic and resistant to degradation. This requirement involves not only the composition of the biomaterials but also their form, internal structure, surface characteristics, and overall design, which should be compatible with the properties of the tissues or structures they are intended to replace. Surface biocompatibility is particularly crucial as it represents the primary interface with the living organism. Therefore, the exposed surfaces must be meticulously engineered to match the chemical, physical, biological, morphological, and medical attributes required for effective integration. By applying suitable surface modification techniques, it is possible to preserve the desired properties of the bulk biomaterials while optimizing specific surface characteristics for various clinical applications [47].

While animal experiments are relatively expensive and require long experimentation periods, cell culture methods can be performed at lower costs, are relatively faster and easier to conduct, and can be easily reproduced. A wide range of in vitro tests have been developed in recent years to evaluate the biocompatibility of various biomaterials. The MTT assay {3-(4,5-dimethylthiazol-2-yl)-2,5-diphenyltetrazolium bromide} is one such in vitro test, which is a sensitive, quantitative, and reliable colorimetric assay that measures cell viability, proliferation, and activation. In living cells, yellow, water-soluble MTT is reduced to a dark-blue formazan product through mitochondrial dehydrogenase enzyme. The amount of formazan produced is directly proportional to the number of viable cells present. Therefore, measuring the optical density (OD) helps determine the amount of formazan produced and thus the number of viable cells present.

For the interpretation of results, the following formula is used:

%cell viability = (OD negative control − OD blank)/(OD positive control − OD blank) × 100, where positive control = cells + compound + MTT + MTT solvent; negative control = cells + MTT + MTT solvent; and blank = medium (complete growth) + MTT + MTT solvent.

The negative control (cell control) and the samples (positive control) were processed in four wells for each compound concentration, and the arithmetic mean of the optical density readings, measured at a wavelength of 570 nm, was calculated at the end.

The results of cell viability determined using the MTT assay led us to conclude the following: (1) the cytotoxicity of the tested compound was dose-dependent; (2) cell viability was lowest in the culture wells, with concentrations of 200 mg/mL of extract for both cell lines and increased proportionally with dilution; (3) regarding the exposure time of the compound on the two cell lines, it was observed that cell viability decreased from 24 to 48 h for fibroblasts, whereas the same trend was not observed for mesenchymal cells (Table 3).

### 2.3. Surgical Intervention

All the procedures were approved by the Ethics Committee of Cantacuzino National Military Medical Institute for Research and Development, A7440/07.10.2021, in accordance with the European Convention for the protection of vertebrate animals used for experimental or other scientific purposes (Strasbourg 18.03.1986) and The Directive 2010/63/UE.

**Figure 7:**
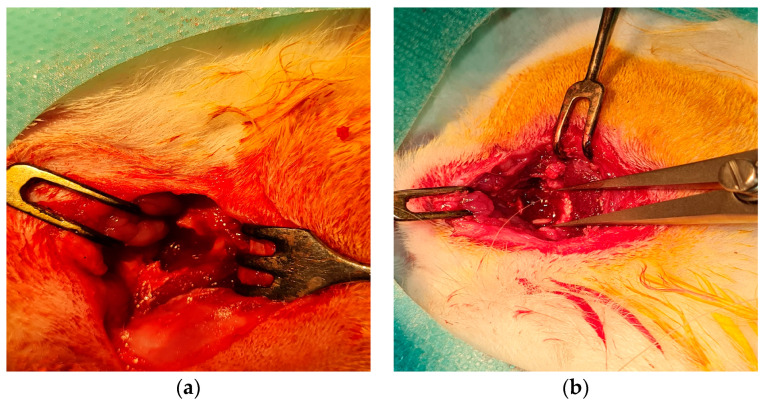
Intra-operatory aspect: (**a**) dissection of the periosteum and creation of the osseous defect; (**b**) the 1 cm diameter osseous defect preformed in the inferior orbital wall.

**Figure 8:**
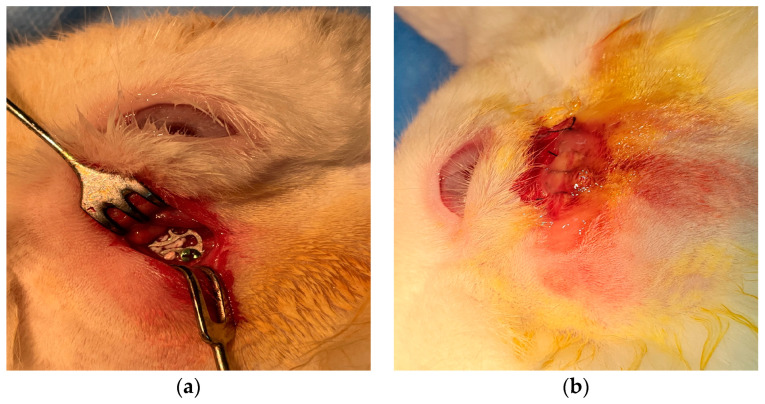
Intra-operatory aspect: (**a**) the orbital implant was positioned within the defect area and sutured to the periosteum; (**b**) orbital periosteum sutures with 9-0 vicryl.

Five male rabbits (*Oryctolagus cuniculus*), approximately 12 months old, were weighed and subsequently anesthetized. The anesthesia protocol involved an initial intramuscular administration of 0.15 mL/kg body weight Xylazine (Xylazine Bio, Maravet Animal Health), followed by an intravenous administration of 0.1 mL/kg body weight ketamine (Ketaminol10, MSD Animal Health) after 10 min. To achieve effective local hemostasis, 0.15 mL of 1/100,000 adrenaline was injected into the lateral canthus and the inferior conjunctival cul-de-sac. An incision was made along the skin around the infraorbital rim, followed by the dissection of the orbicularis oculi muscle, allowing adequate exposure of the infraorbital rim. The periosteum was then dissected and meticulously separated from the bone to avoid any tearing. A circular bone defect with a diameter of 1 cm was created at the center of the exposed area using a stomatologic trephine (Figure 7). The nanostructured implant was positioned within the defect and secured to the orbital periosteum using 9-0 vicryl sutures (Figure 8). The skin was closed using 5-0 vicryl sutures. A 0.3% tobramycin and 0.1% dexamethasone ointment (Tobradex, Alcon, Fort Worth, TX, USA) was applied for 5 days.

Postoperative ophthalmological assessments were conducted daily using slit lamp evaluation for the first 10 days. Subsequently, evaluations were performed every third day for a period of one month. The bone fragment containing the implant was harvested two months post-surgery through a similar surgical procedure, involving a drilling process following the opening of the orbital septum to facilitate bone extraction. The harvested bone was then placed in 10% formaldehyde and subsequently processed for paraffin embedding and histopathological/immunohistochemical analysis.

### 2.4. Histological and Immunohistochemistry Results

Histopathology slides were prepared to examine the harvested tissue. The examined fragments varied in size from 1 to 6 mm, containing, in some cases, a predominant component of the amorphous tissue used experimentally. The usual hematoxylin–eosin (H&E) staining was used for the initial evaluation. Small tissue fragments harvested through drilling from the sub-orbital area of the rabbit were analyzed and reported alongside fragments of an amorphous, synthetic material, i.e., the alloplastic material.

**Figure 9:**
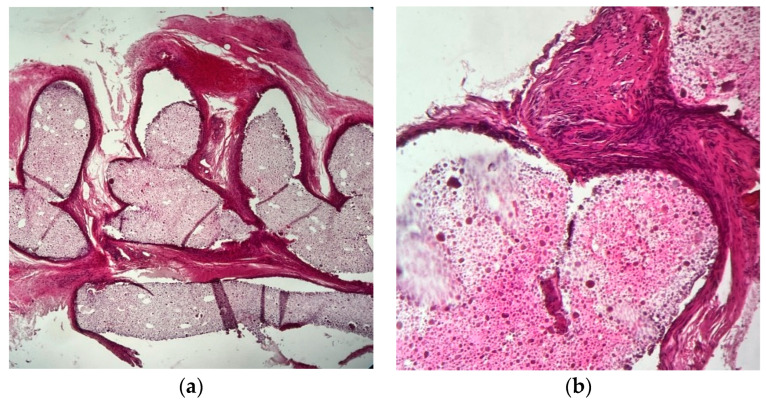
H&E staining: (**a**) 4× magnified image of the studied material filling the natural spaces; (**b**) 20× magnified image of a detail of the examined allogenic material and its relation to adjacent tissue. Mature granulation tissue with fibroblast and capillaries are easily seen, which suggests a vital regenerative response.

The material that was used as the object of our study has a granular, non-homogeneous, amorphous appearance, and it is capable of being insinuated into natural or surgically induced spaces, filling them (Figure 9).

**Figure 10:**
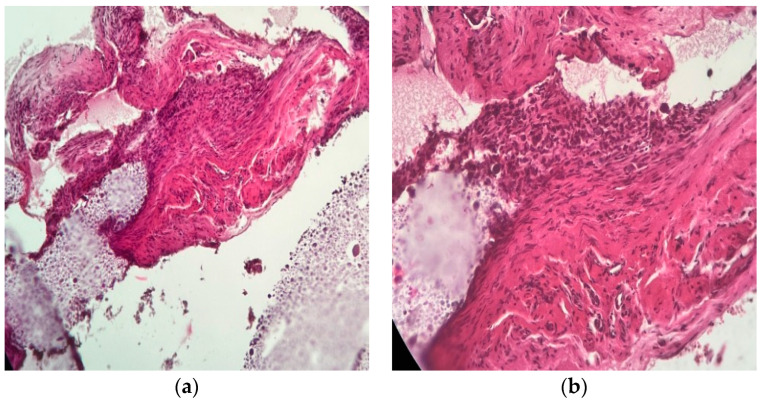
H&E staining. In the center of the image, a fibro–conjunctival–vascular tissue can be identified, which, upon intimate contact with the material proposed to be evaluated, shows an abundant proliferation of fibrocytes. In the upper left corner of the image, numerous small capillaries can be seen: (**a**) 10× magnified image of the studied material filling the natural spaces; (**b**) 20× magnified image of a detail of the examined allogenic material and its relation to adjacent tissue. Mature granulation tissue with fibroblast and capillaries are easily seen, which suggests a vital regenerative response.

**Figure 11:**
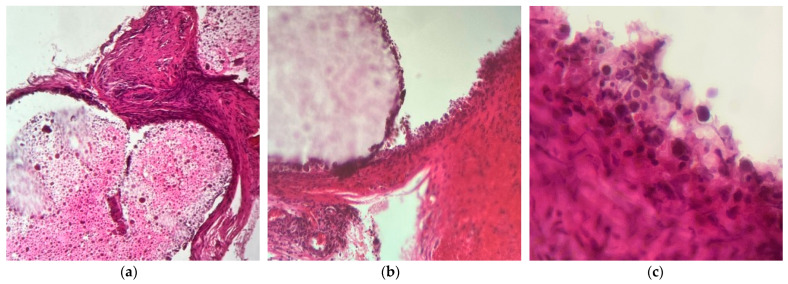
H&E staining: (**a**) 10× magnified image of the abundant hypercellular reaction predominantly composed of fibroblasts, with elongated and wavy nuclei alongside rare vessels and inflammatory cellularity of the lymphomonocytic series between two segments of allograft material. (**b**) Images, 20× magnified—on the upper left, one can see the material with a macrophage border on the edge (this is proof of its participation in re-epithelialization); in the middle, one can see the intercepted fibrous tissue that additionally shows a fibroblastic proliferation; to the lower left, one can identify 4 vascular lumens with red blood cells, incorporated in a connective tissue with inflammatory elements and fibroblasts, which are compatible with a granulation tissue. (**c**) A 40× magnified image of a detail of the reactive cellularity in the examined material. Macrophages that have phagocytosed the studied material can be detected along with rare lymphocytes and an immature collagen matrix. Polymorpho-nucleates are not identified.

In most cases, its presence is accompanied by a fibroblastic reaction (additional IHC studies are needed to discriminate between fibroblasts, rhabdomyoblasts, and endothelial precursors), devoid of acute inflammation (no PMNs were detected in any of the preparations, indicating the absence of tissue-destructive reactions accompanied by necrosis or reactive acute inflammation due to bacterial contamination). Macrophages were frequently observed (Figure 10 and Figure 11).

Histopathology slides were prepared to examine the harvested tissue. The examined fragments varied in size from 1 to 6 mm, containing, in some cases, a predominant component of the amorphous tissue used experimentally.

The results have proven the following findings:

- The inoculated material was accepted by the host organism and did not demonstrate any suggestive elements for necrosis or infection. No multinucleated cells or acute phase cells were identified on any of the slides.
- The allograph material was partially phagocytosed, with the activated macrophages stimulating an abundant fibroblastic reaction through chemotaxis and inflammatory mediators.
- Neovascular formation was present in all cases, indicating an active repair process that, in the absence of intervention, would have continued towards healing.
- The interface between the material and the host tissue demonstrates, through the presence of young collagen, the supporting role of the material in the production of extracellular matrix by the organism.

**Figure 12:**
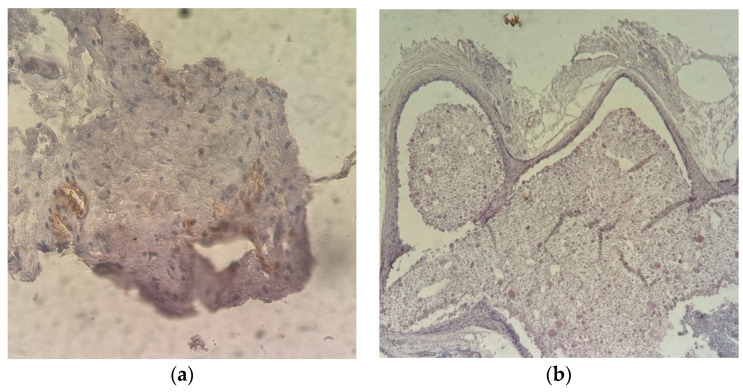
Immunohistochemical staining. In the center of the image, a fibro–conjunctival–vascular tissue can be identified which, upon intimate contact with the evaluated material, shows an abundant proliferation of fibrocytes. In the upper left corner of the image, numerous small capillaries can be seen. (**a**) CD 31; 40× magnified image of positivity in capillaries and in small groups of nucleated cells, compatible with leucocytes. (**b**) Surrounding tissue of the allogenic material presents rare capillaries with endothelial cells positive for CD31.

**Figure 13:**
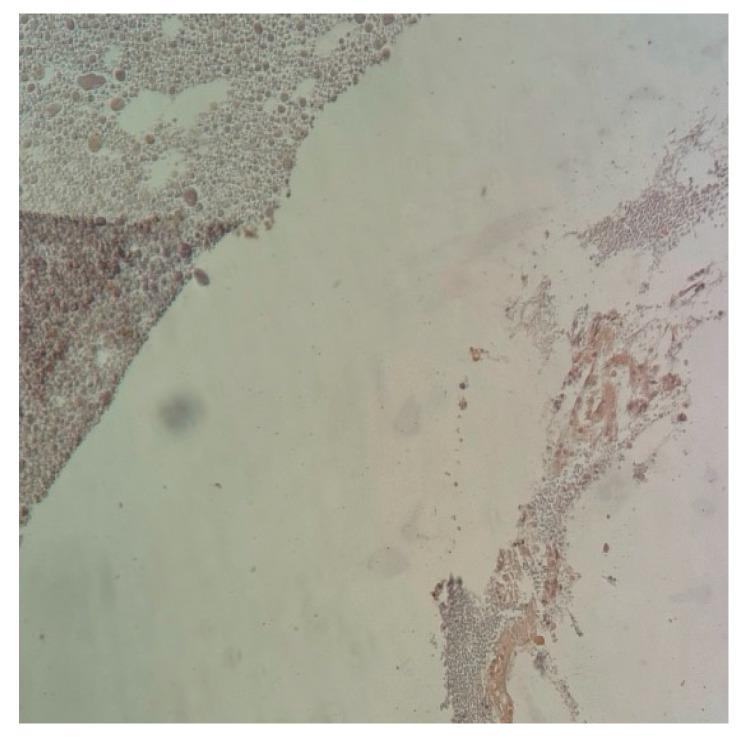
Immunohistochemical staining; 40× magnified image. On the second lot, in the granulation tissue adjacent to the studied material, rare nucleated (leukocytes) and anucleated (platelets, red blood cells) cells, positive for CD34, can be observed.

The IHC tests performed used CD31 and CD34 antibodies. CD31 is a cell adhesion molecule necessary for leukocyte transendothelial migration in inflammatory conditions and is a marker of the macrophage-mediated phagocytosis of apoptotic leukocytes. Its expression is membranous, on the edge between the endothelial cells in contact with platelets, neutrophils, or leukocytes. In the first examined group, a perivascular expression was observed in the intercepted capillaries and in rare inflammatory cells, i.e., nucleated (PMNs, macrophages) or anucleated cells (platelets) (Figure 12 and Figure 13).

CD34 is a membrane-expressed adhesion molecule on hematopoietic progenitor cells and on the endothelium of small vessels.

The present study is not without limitations. These include the following: the absence of a control subject whose healing occurred naturally without the intervention of an external material; the small size of the biopsies did not allow histological relationships to be established between the induced lesion, the experimentally used reparative material, and the obtained callus; the lack of decalcification was an obstacle for evaluation of the bone response; the very small fragments, with a number of <100 cells; the lack of an internal control limited the quantitative interpretation of the sample of interest in IHC.

Histological analyses revealed the successful integration of the implant into the host tissue, as evidenced by fibrovascular ingrowth within the pores of the implant. In conclusion, our engineered 3D-printed HAp-COL demonstrates stability and biocompatibility, making it a viable option for the safe reconstruction of orbital wall fractures.

Bone formation follows a natural process that consists of two stages: primary and secondary osteogenesis [24,48]. Primary osteogenesis is the process of bone development that starts from existing cartilage, called endochondral osteogenesis. In this process, hydroxyapatite (HA) crystals are formed in a disorganized manner inside a proteoglycan matrix, culminating in the production of woven bone. At this moment, there is no direct correlation between HA crystals and collagen. Therefore, initial osteogenesis is often not taken into account when trying to reproduce the bone production process using collagen. Secondary osteogenesis is characterized by the transformation of primary woven bone into a more structured form by the incorporation of tiny HA crystals inside collagen fibers. This process is referred to as intrafibrillar mineralization [49]. The process of bone production requires the existence of non-collagenous proteins (NCPs), such as osteonectin and osteocalcin, a small number of mineral ions, and the movement of extracellular fluid (ECF) inside the bone structure [50]. NCPs play a vital role in the mineralization process by binding calcium and phosphate ions found in the ECF [51]. This binding helps in the creation of a liquid amorphous calcium phosphate phase called polymer-induced liquid precursor (PILP). The high attraction between NCPs and collagen, together with the liquid-like properties of PILP, enables the calcium phosphate precursor to penetrate collagen fibrils. The amorphous calcium phosphate phase undergoes dehydration and later converts into a more thermodynamically stable crystalline form inside the collagen fibers [52].

In this context, several studies have suggested that the use of a continuous perfusion flow (referred to as dynamic intrafibrillar mineralization) could more accurately mimic the mineralization observed in physiological models [24]. However, to our knowledge, there is currently no study that has demonstrated the effectiveness of this model in vivo, and further research is needed in this area.

## 3. Materials and Methods

### 3.1. Hydrothermal Synthesis of Hydroxyapatite–Collagen (HAp-COL) Hybrid Powder

Hydroxyapatite–collagen powder was obtained through the hydrothermal synthesis method starting from calcium nitrate, ammonium dihydrogen phosphate, and hydrolyzed collagen, dissolved in distilled water, under magnetic stirring. Then, 25% ammonia was gradually added to the solution until an alkaline pH (pH = 10) was reached. The resulting suspension was transferred to the Teflon vessel of the Berghof autoclave for hydrothermal synthesis under controlled temperature and pressure conditions (100 °C and 10^4^ Pa). The obtained precipitate was separated from the aqueous phase in the suspension through vacuum filtering and washing, until pH = 7, followed by spray-drying using Lab PLANT equipment. A white, granulated powder based on hydroxyapatite and collagen (HAp:COL mass ratio = 4:1) was obtained and then processed for 2 types of coatings on titanium mesh. These were further tested in vitro and in vivo as possible orbital implants: (i) HAp-COL powder was pressed, sintered, and used as a “target” to coat the Ti mesh through the EB-PVD method; (ii) HAp-COL powder was mixed with commercial dispersants and binders to prepare a hybrid paste which serves as raw material for 3D printing of HAp-COL structures on Ti mesh or as a stand-alone 3D structure.

### 3.2. Three-Dimensional Printing of HAp-COL Structures on Ti Mesh

HAp-COL hybrid powders, obtained through the hydrothermal method, were further used for the preparation of pastes; these represent the raw materials that are inserted into the syringe of a 3D printer and serve to print 3D structures based on HAp-COL.

In order to obtain printable pastes, these hybrid powders were mixed with commercial dispersants (Baymedix FD 103, Covestro, solution from Bayer AG of Leverkusen, Germany) and binders (polyvinyl alcohol) in the Thinky-ARE 250 centrifugal planetary mixer for 6 min at 2000 rpm for homogenizing and for 4 min at 2000 rpm for degassing. The mixer allows the preparation of pastes directly in the syringe of the 3D printing equipment, thus avoiding the formation of air bubbles during syringe filling. The obtained pastes, comprising 53% HAP-COL powder, 22% Baymedix FD 103, and 25% polyvinyl alcohol (PVA), were used for the fabrication of 3D structures through an extrusion-based 3D printing technique using a 3D BIOPLOTTER EnvisionTEC Starter Series printer.

The 3D structures were obtained using extrusion-based 3D printing with a 3D-BioPlotter Starter Series (EnvisionTEC GmbH, from Gladbeck, Germany) system, connected to a computer for *STL files import and for controlling of the 3D-Bioplotter. After the *STL files were imported and the desired number of layers was created, the model of the 3D structure (distance between strands, orientation of strands, pattern, and distance from the substrate) was selected, the needle diameter was chosen, and the syringe filled with the paste was fixed in the corresponding printing head. The actual printing started after the 3D printer was calibrated according to the working instructions.

The working parameters were as follows:

- For the layers deposited on the titanium mesh:

(a) Parallelepiped with dimensions of 20 × 20 × 5 mm^3^, needle diameter = 0.4 mm, distance between strands = 0.8 mm, angle between layers (orientation of strands) = 0°/45°/90°, printing of the paste on Ti mesh pieces of 5 × 5 mm in size, placed next to each other and framed in parallelepiped material.

(b) Parallelepiped with dimensions of 60 × 5 × 0.96 mm^3^, needle diameter = 0.4 mm, distance between strands = 0.8 mm, angle between layers (orientation of strands) = 0°/45°/90°, with or without contour.

Printing on the titanium mesh involved the use of a double adhesive tape for fixing the metal mesh to the printing surface. On the surface of the titanium mesh, 3 layers of hydroxyapatite–collagen paste were deposited through extrusion-based 3D printing, so that the thickness of the deposited layer was 0.96 mm.

### 3.3. Physicochemical, Structural, and Morphological Characterization of the Hybrid Powder

The hybrid powder, obtained as described above, was characterized through the following physicochemical and morphological analysis methods:

Quantitative chemical analysis: The Ca content was determined using the flame atomic absorption spectrometry (FAAS) method; the P content was determined through the inductively coupled plasma optical emission spectrometry method (ICP-OES).

FT-IR spectroscopy analysis: The presence of functional groups (chemical structure of the powders) was identified through Fourier transform infrared spectroscopy (FT-IR), using an ABB MB 3000 FT-IR spectrometer (ABB from Zurich, Switzerland), equipped with an EasiDiff device for powders. The solid sample is mixed with KBr so that its concentration in the mixture is 1% gravimetric. The mixture thus obtained is ground for 10–15 min to obtain fine, homogeneous particles. For data acquisition, 64 scans were made at a resolution of 4 cm^−1^, working in the range of 550–4000 cm^−1^ in transmittance mode. The processing of experimental data was carried out using the Horizon MB^TM^ FT-IR software (Software Version 3.4).

X-ray diffraction analysis (XRD): The obtained powder was investigated using XRD to determine the crystalline phases. For the measurements, a Bruker-AXS D8 ADVANCE Bragg–Brentano diffractometer with a radiation source (Cu) and SOL X detector in vertical geometry θ–θ, equipped with BRUKER-AXS software (Software Version 5.1), was used. The diffraction spectra were acquired in the angular range 6–70°, in continuous mode, with a step of 0.02°. Data processing was performed with the help of the DIFFRAC. EVA VER.5 2019 program from the DIFFRAC. SUITE. EVA software (Software Version 7) package and the ICDD PDF-5 + 2024 database.

Electron microscopy (SEM) analysis: The study of particle morphology was carried out with an FEI Quanta 250 electron microscope in high-vacuum (HV) (ESEM) working mode, using a secondary electron detector (ETD), a backscattered electron detector (CBS), and a detector for energy-dispersive spectroscopy (EDS).

### 3.4. Cytotoxicity Testing of HAp-COL Compound Through In Vitro Cell Culture Studies

For the cytotoxicity test of the HAp-COL compound (powder), two cell lines were utilized: a fibroblast line (3T3) and a mesenchymal stem cell (MSC) line, derived from adipose tissue (ATCC, PCS-500-011). The 3T3 fibroblast cell line was cultured in 25 cm^2^ cell culture flasks using Dulbecco’s Modified Eagle’s Medium (DMEM) supplemented with 10% fetal bovine serum (FBS) and 50 μg/mL gentamicin. The MSC line was cultured in 25 cm^2^ cell culture flasks using complete growth medium (mesenchymal stem cell basal medium supplemented with the mesenchymal stem cell growth kit). For each cell line, cells from two 25 cm^2^ flasks were trypsinized with 0.025% trypsin–EDTA (for fibroblasts) and 0.05% trypsin and 0.02% EDTA (for MSC) and centrifuged; then, the pellet was resuspended in 5 mL of specific complete growth medium for each cell line. Initial cell counting was performed using 1:1 Trypan Blue staining (50 μL cell suspension + 50 μL Trypan Blue). The fibroblast cell suspension had a concentration of 1.17 × 10^6^ cells/mL, while the MSC suspension had a concentration of 5.7 × 10^5^ cells/mL.

The test compound, in powder form, was weighed and placed in sterile glass bottles with each of the two complete growth media, specific to each cell line, and incubated for 72 h at 37 °C, 5% CO_2_. The weight/volume ratio was 200 mg/mL, according to the ISO Standard. After incubation, the 200 mg/mL stock solution was diluted into binary dilutions (100 mg/mL, 50 mg/mL, and 25 mg/mL). For the cytotoxicity test, cell suspensions were cultured in two 96-well flat-bottom microplates, with 200 μL/well (3.34 × 10^5^ cells/well for fibroblasts and 1.14 × 10^4^ cells/mL for MSC) and incubated in a CO_2_ incubator (5%) at 37 °C for 24 h.

The next day, the medium was changed as follows:

- In the blank wells, only 200 μL of complete growth medium was added.
- In the cell control wells, the medium was removed and 200 μL of fresh complete growth medium was added.
- In the wells with the test compound, the medium was removed and 200 μL of fresh medium containing different concentrations of the compound (200 mg/mL, 100 mg/mL, 50 mg/mL, and 25 mg/mL) was added.

Two 96-well culture microplates were prepared, each containing the two cell lines and the test compound (in serial binary dilutions); one plate was incubated for 24 h and the other was incubated for 48 h at 37 °C, 5% CO_2_. For each cell line, the blank was allocated in duplicate, and the cell control and the test compound were allocated in four wells for each dilution.

After 24 and 48 h, the 3-(4,5-dimethylthiazol-2-yl)-2,5-diphenyltetrazolium bromide (MTT) assay was performed to measure the conversion of MTT into a colored product using living cells. For this test, we used the MTT-based cell growth determination kit (Sigma, St. Louis, MO, USA), which includes the MTT solution (5 mg/mL MTT in RPMI-1640 without phenol red) and the MTT solvent (0.1 N HCl in anhydrous isopropanol).

The microplate was removed from the incubator, 20 μL of MTT solution was added to each well (10% of the medium volume), and the plate was incubated for 4 h at 37 °C, in the dark, with CO_2_. After 4 h, the microplate was taken out of the incubator, the medium was removed, and 200 μL of MTT solvent was added to each well. The optical density was read at a wavelength of 570 nm, within one hour of adding the solvent, using a multimodal reader (EnSight™ Multimode Microplate Reader, PerkinElmer, Thane, Maharashtra).

### 3.5. Histopathological Material and Methods

For in vivo analysis, histopathological and immunohistochemistry tests were performed using the harvested tissue obtained through a drilling process, represented using small fragments of bone and adjacent tissue containing the implant. Tissue fragments were directly placed in 10% formalin and subsequently processed for paraffin embedding and histopathological/immunohistochemical analysis. Considering the lack of experience regarding the reaction between HAp-COL compound and ethylenedinitrilo tetraacetic acid (EDTA), the usual step for decalcification was avoided. Each fragment was embedded in a separate block, resulting in six distinct blocks from five subjects, in which the collected material was fully included.

The FFPE tissue samples were cut into sections of 4 µm thickness using a rotary microtome (Amos Scientific, Melbourne, Australia). The sections were deparaffinized in 3 successive xylene changes, each lasting 10 min. Subsequently, the sections were rehydrated in graded ethanol series down to 70%, washed in water, and stained with Mayer’s hematoxylin (Bio-Optica, Milan, Italy). After treatment with lithium carbonate, the slides were differentiated using a 0.5% HCl solution in 70% ethanol and stained with a 1% aqueous solution Eosin Y (Bio-Optica, Milan, Italy). To complete the process, the slides were dehydrated using a series of ethanol solutions with increasing concentrations and clarified with xylene. Finally, all slides were mounted with coverslips using CV Mount (Leica Biosystems, Wetzlar, Germany) as the mounting medium. The hematoxylin–eosin (H&E)-stained sections were analyzed using bright-field microscopy (Zeiss LSM980 from Carl Zeiss Microscopy, Oberkochen, Germany) independently by two researchers, in a blind manner.

### 3.6. Immunohistochemistry Material and Methods

Additionally, tissue samples were sagittally cut at a thickness of 4 μm using a rotary microtome (Amos Scientific, Melbourne, Australia) and stained for CD31 and CD34. Briefly, sections were deparaffinized in 3 successive xylene changes of 10 min each, rehydrated in a graded ethanol series down to 50%, and washed in running tap water. A heat-mediated antigen retrieval was performed using a sodium citrate buffer (pH 6.0). Next, the sections were blocked for 2 h at room temperature in a humidity chamber with 10% normal serum (Vector Laboratories Inc., Newark, CA, USA) with 1% BSA in TBS; then, they were incubated overnight at 4 °C with the anti-CD31 primary monoclonal antibody (JC/70A) (Novus Biologicals, Minneapolis, MN, USA, catalog no. NB 600-562), diluted 1:25 in TBS with 1% BSA, and the anti-CD34 primary monoclonal antibody (MEC14.7) (GeneTex, Irvine, CA, USA, catalog no. GTX28158), diluted 1:250 in TBS with 1% BSA.

For IHC immunostaining, endogenous peroxidase was blocked with 3% H_2_O_2_ followed by incubation with the HRP-conjugated secondary antibody (rabbit anti-mouse IgG H&L (HRP), (Abcam from Cambridge, UK), catalog no. ab6728; goat anti-rat IgG H&L (HRP), Abcam, catalog no. ab97057), diluted 1:200 in TBS with 1% BSA. The staining was developed using DAB chromogen (DAB Substrate Kit, Thermo Fisher Scientific, Waltham, MA, USA, catalog no. 34002); the sections were counterstained with hematoxylin and subsequently dehydrated, cleared, and mounted with CV Mount (Leica Biosystems, Wetzlar, Germany). The samples were examined using a Zeiss LSM980 (Carl Zeiss, Oberkochen, Germany) confocal microscope equipped with a 40×/1.3 plan-Apochromat oil differential interference contrast (DIC) objective lens, and the images were captured using Zen Blue software (Software Version 3.4).

## 4. Conclusions

A new approach for orbital wall reconstruction was described in this paper, combining the chemical synthesis of hydroxyapatite–collagen hybrid powder—a traditional bone substitute material due to its similarity with natural bone—with an additive manufacturing technique. This technique is an emergent technology which enables the fabrication of tridimensional structures with controlled porosity. The preparation included the coating of a Ti mesh with 3D-printed structures to ensure the mechanical resistance of the implant and its fixation on the orbital wall.

Hydroxyapatite–collagen hybrid powders were prepared through the hydrothermal method in high-pressure conditions and further used as a coating for a titanium mesh, with the aim of repairing and reconstructing the orbital wall in a rabbit animal model. The hydrothermal approach to obtain HAp-COL has various benefits, including the use of an aqueous reaction media, the production of crystalline and pure materials with a controllable shape, and low energy usage. HAp-COL coatings were prepared through the 3D printing of HAp-COL structures directly on Ti mesh; these were then used as orbital implants. In vitro and in vivo tests showed that the 3D-printed HAp-COL structures had high biocompatibility, and we observed good integration of the implant in the bone. The data presented in this study provide compelling evidence to demonstrate that our engineered 3D-structured HAp-COL may be safely considered for use in the reconstruction of orbital wall fractures.

## Figures and Tables

**Table 1:** Results obtained through chemical analysis.

Crt No.	Sample Code	Ca, %	P, %
1	HAp-COL	35.94	16.50

**Table 2:** Crystalline phases and crystallization systems of the compounds identified with the references in the XRD database.

Sample Code (Powder)	Crystalline Phase Identified Using XRD	Chemical Formula	Identified PDF Card	Crystallization System	S-Q (%) Rietveld
HAP-COL	Hydroxyapatite 1	Ca_5_(PO_4_)_3_(OH)	PDF 01-082-2956	Hexagonal	31.63%
	Hydroxyapatite 2	Ca_5_(PO_4_)_3_(OH)	PDF 00-071-5048	Hexagonal	68.37%

**Table 3:** Cell viability (%) at 24 and 48 h.

Tested Sample	Viability (%)			
	24 h		48 h	
	3T3	MSC	3T3	MSC
Hybrid 200 mg/mL	80.69	66.63	79.10	75.47
Hybrid 100 mg/mL	82.45	77.27	84.21	81.34
Hybrid 50 mg/mL	83.58	85.04	89.76	84.65
Hybrid 25 mg/mL	89.24	91.35	91.79	87.08

## Data Availability

The data underlying the results presented in this paper are not publicly available at this time but may be obtained from the authors upon reasonable request.
